# Pose Estimation of Sweet Pepper through Symmetry Axis Detection

**DOI:** 10.3390/s18093083

**Published:** 2018-09-13

**Authors:** Hao Li, Qibing Zhu, Min Huang, Ya Guo, Jianwei Qin

**Affiliations:** 1 Key Laboratory of Advanced Process Control for Light Industry, Ministry of Education, Jiangnan University, Wuxi 214122, China; 6161905027@vip.jiangnan.edu.cn (H.L.); huangmin2004@jiangnan.edu.cn (M.H.); Guoy@jiangnan.edu.cn (Y.G.); 2 USDA/ARS Environmental Microbial and Food Safety Laboratory, Beltsville Agricultural Research Center, Bldg., 303, BARC-East, 10300 Baltimore Ave., Beltsville, MD 20705-2350, USA; jianwei.qin@ars.usda.gov

**Keywords:** pose estimation, symmetry axis, point cloud, sweet pepper

## Abstract

The space pose of fruits is necessary for accurate detachment in automatic harvesting. This study presents a novel pose estimation method for sweet pepper detachment. In this method, the normal to the local plane at each point in the sweet-pepper point cloud was first calculated. The point cloud was separated by a number of candidate planes, and the scores of each plane were then separately calculated using the scoring strategy. The plane with the lowest score was selected as the symmetry plane of the point cloud. The symmetry axis could be finally calculated from the selected symmetry plane, and the pose of sweet pepper in the space was obtained using the symmetry axis. The performance of the proposed method was evaluated by simulated and sweet-pepper cloud dataset tests. In the simulated test, the average angle error between the calculated symmetry and real axes was approximately 6.5°. In the sweet-pepper cloud dataset test, the average error was approximately 7.4° when the peduncle was removed. When the peduncle of sweet pepper was complete, the average error was approximately 6.9°. These results suggested that the proposed method was suitable for pose estimation of sweet peppers and could be adjusted for use with other fruits and vegetables.

## 1. Introduction

Fruit harvesting is an important part of the entire production process of fruit farming. In the production process, prevalent harvesting methods are still based on high-cost, -intensity, and -risk manual harvesting, in which the labor force employed accounts for 33% to 50% of the total labor force [1]. In the Washington State alone, the harvesting cost for handpicking apple is approximately $1150 to $1700 per acre per year [2]. A total of $21 million was used for personal injury compensation related to manual harvesting between 1996 and 2001 in the USA [3]. More importantly, the agricultural labor force worldwide exhibits a declining trend with aging population, which aggravates the problem of labor shortage and labor cost increase, and eventually affects sustainable fruit cultivation development [4]. Thus, automated harvesting systems must be developed to meet the increasing labor demand, to decrease human risks of injuries in orchards, and to decrease the harvesting cost by saving time, money, and energy to benefit producers and consumers [5].

Since Schertz and Brown [6] first introduced the concept of automatic harvesting as an alternative to mechanical harvesting, the development of an automated crop harvesting system has been a crucial topic in the field of agricultural engineering. After approximately 50 years of development, many automatic harvesting machines based on various working principles and structural forms were studied and proposed [7,8,9]. A harvesting robot based on machine vision technology is the most important fruit harvesting machine because it can automatically obtain a variety of information (such as plant structure, fruit location, and surrounding environment), and thus, facilitate the appropriate picking action to reduce the damage to the fruit and plant.

Fruit detection in plants provides fundamental information for developing harvesting robots. The accuracy in detection of a fruit is easily influenced by uncertain and variable lighting conditions in the field; variable and complex canopy structures; and varying colors, shapes, and sizes of fruits [10]. Numerous methods were established to improve the accuracy of fruit detection in similar outdoor environments. These methods mainly focus on the following: (1) acquiring an image using different types of visual sensors (i.e., black/white (B/W), colored, spectral, and thermal cameras); (2) extracting color, geometric, and texture features from the acquired images using different imaging analysis techniques; (3) and identifying fruit object from the whole images using different machine learning methods with different extracted features. Edan et al. [11] used a B/W camera to detect melons using the intensity levels of reflectance and texture and analyzing shape; 82–88% of the fruits were detected under real field conditions. Cohen [12] identified 85% of the apples using combined color and texture analyses with a standard color charge-coupled device (CCD) camera. Safren et al. [13] used principle component analysis to reduce high-dimensional data from hyperspectral camera. Homogeneous objects were extracted and classified, and morphological operations, watershed analysis, and blob analysis were then conducted. The integration of these methods led to a fruit segmentation accuracy of 88.1%.

Fruit localization in trees is another important ability of harvesting robots, which locate the fruit in a three-dimensional (3D) coordinate system, and are used to guide the manipulator and end-effector move to the desired position for picking action. Mehta et al. [14] calculated the citrus position detected by a single camera to the robot base; they reported that the accuracy of estimating the position was approximately 15 mm. Bulanon et al. [15] used a color camera and a laser sensor, which were mounted together in a cylindrical manipulator controlled by a visual servo method. The target fruit center was aligned at the center of image by visual servoing, and a laser range sensor measured the distance to the fruit. The accuracy of the system for estimating the distance to the fruit was ±3 mm. Plebe and Grasso [16] used stereo cameras to locate oranges in a 3D coordinate system by stereo matching based on artificial neural networks (ANNs). Gongal et al. [17] used a time-of-flight (TOF) camera to determine the 3D coordinates of the fruit in apple trees.

After the detection and localization of the fruit, automatic harvesting robots implement the detachment action. Studies suggested that the efficiency of fruit detachment can be improved if the fruit is rotated or twisted in a particular manner relative to the orientation of the fruit and peduncle [18,19]. Researchers attempted to detach the fruit and peduncle through peduncle detection. Sa et al. [20] utilized color and geometry information acquired from a red/green/blue-depth (RGB-D) sensor coupled on a support vector machine for peduncle detection. The performance of the proposed method was evaluated using qualitative and quantitative results (the area under the curve (AUC) of the detection precision–recall curve). The method achieved an AUC of 0.71 for peduncle detection of field-grown sweet peppers. Eizentals and Oka [21] presented a peduncle estimation algorithm for automatic harvesting of Japanese green pepper. In the laboratory test, the mean total error for the affine transformation was less than 25 mm in 42 of 49 positions, less than 20 mm in 28 of 49 positions, and less than 15 mm in 19 of 49 positions. However, the direct detection of peduncle with a machine vision system remains challenging because it is small and often occluded by the fruit. Future research in machine vision systems should focus on estimating the orientation of the fruit and the orientation and location of the peduncle. Determination of fruit geometric parameters, such as symmetry of the shape, can provide means to estimate the direction of the fruit [5]. However, few studies investigate pose estimation based on the symmetry axis of the fruit.

**Figure 1:**
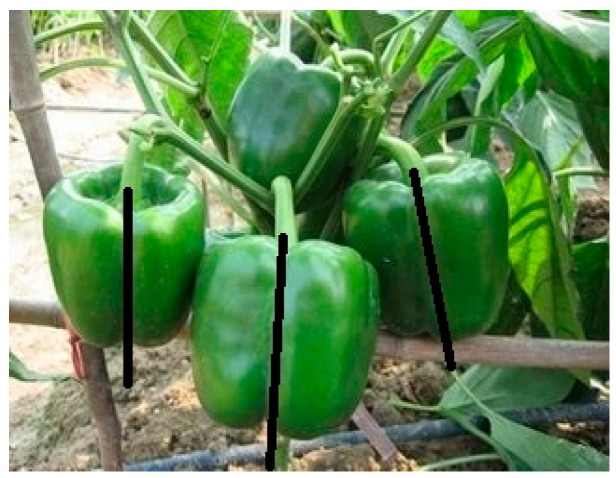
Example of the symmetry axis passing through the peduncle (the axis is manually annotated).

Sweet pepper naturally has a regular shape and a symmetry axis (Figure 1). Hence, the method proposed to estimate the pose of sweet pepper by detecting the symmetry axis is feasible. In contrast to previous methods, the proposed method estimates sweet-pepper posture based on the symmetry axis, regardless of whether the peduncle is small or occluded.

Overall, this study aimed to estimate the pose of sweet peppers and to provide effective guidance for the end-effector of an automatic harvesting robot. The specific goal was to calculate the symmetry axis of sweet pepper.

## 2. Dataset and Methods

### 2.1. Dataset

**Figure 2:**
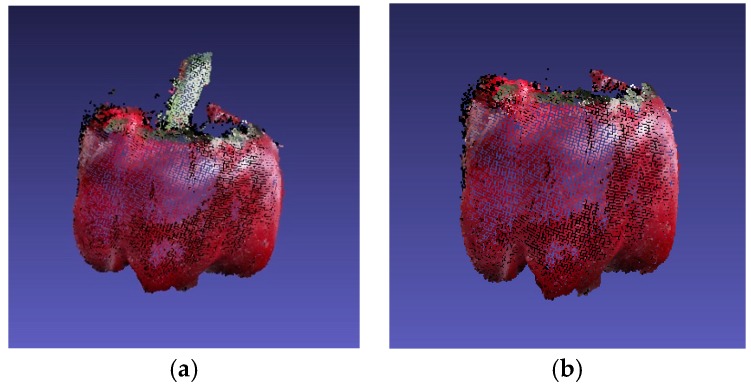
Three-dimensional (3D) pepper point cloud in MeshLab. (**a**) Full pepper; (**b**) sweet pepper with removed peduncle.

The proposed pose estimation algorithm was tested on a set of manually annotated 3D images of sweet pepper and peduncle to evaluate its performance. The dataset obtained from an Intel RealSense SR300 camera was reported by Sa et al. [18]. In their work, the preregistered 3D models of the scene containing the peduncle and sweet pepper were obtained. They also used a statistical outlier remover and a voxel grid down sampler supported from the point cloud library [22] to filter the dataset. The dataset included 27 point clouds with peduncle (Figure 2a) and point clouds with removed peduncle (Figure 2b).

**Figure 3:**
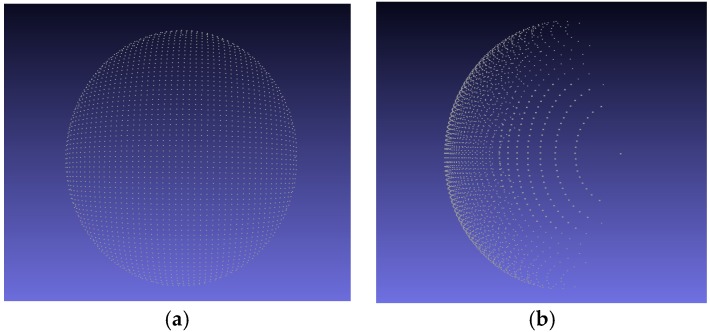
Simulated point cloud 20 cm away from the camera viewed from different perspectives. (**a**) Front view of the simulated point cloud; (**b**) side view of the simulated point cloud.

The real symmetry axis could not be obtained because the sweet-pepper point cloud provided by Sa et al. was not manually calibrated. To verify the accuracy of the algorithm, we simulated a dataset to compare errors between the calculated and real axes. The sweet-pepper point cloud was simulated by the fitted ellipsoid, whose symmetry axis could be manually determined. An ellipsoid was produced as follows:

(1) $$\frac{{\left(x-x_{0}\right)}^{2}}{x_{1}{}^{2}}+\frac{{\left(y-y_{0}\right)}^{2}}{y_{1}{}^{2}}+\frac{{\left(z-z_{0}\right)}^{2}}{z_{1}{}^{2}}=1,$$

where $x_{0}$, $y_{0}$, and $z_{0}$ represent the coordinates of the center of the point cloud; and $x_{1}$, $y_{1}$, and $z_{1}$ represent the half-axis of the point cloud on the *x*-, *y*-, and *z*-axes, respectively. The Intel RealSense SR300 depth camera has a working range of 20–150 cm. As the distance increases, the accuracy of the camera decreases. In the simulation, the depth between the ellipsoid and the viewpoint $z_{0}$ increased from 25 cm to 70 cm with a step of 1 cm; thus, 45 point clouds were tested in each experiment using the proposed algorithm. The values of $x_{0}$ and $y_{0}$ were set to 3 and 5 cm, respectively, to simulate the offset of the point cloud from the camera. To simulate the size of the sweet pepper in space, we set the size of the point cloud to approximately 5 cm × 5 cm × 4 cm ($x_{1}$ = 2.5 cm, $y_{1}$ = 2.5 cm, $z_{1}$ = 2 cm). The depth camera uses KinFu technology, which made an incomplete 3D reconstruction of the sweet-pepper cloud point (the part located opposite the camera was invisible). Therefore, we artificially removed this part from the viewpoint of the ellipsoid (Figure 3).

### 2.2. Method

**Figure 4:**
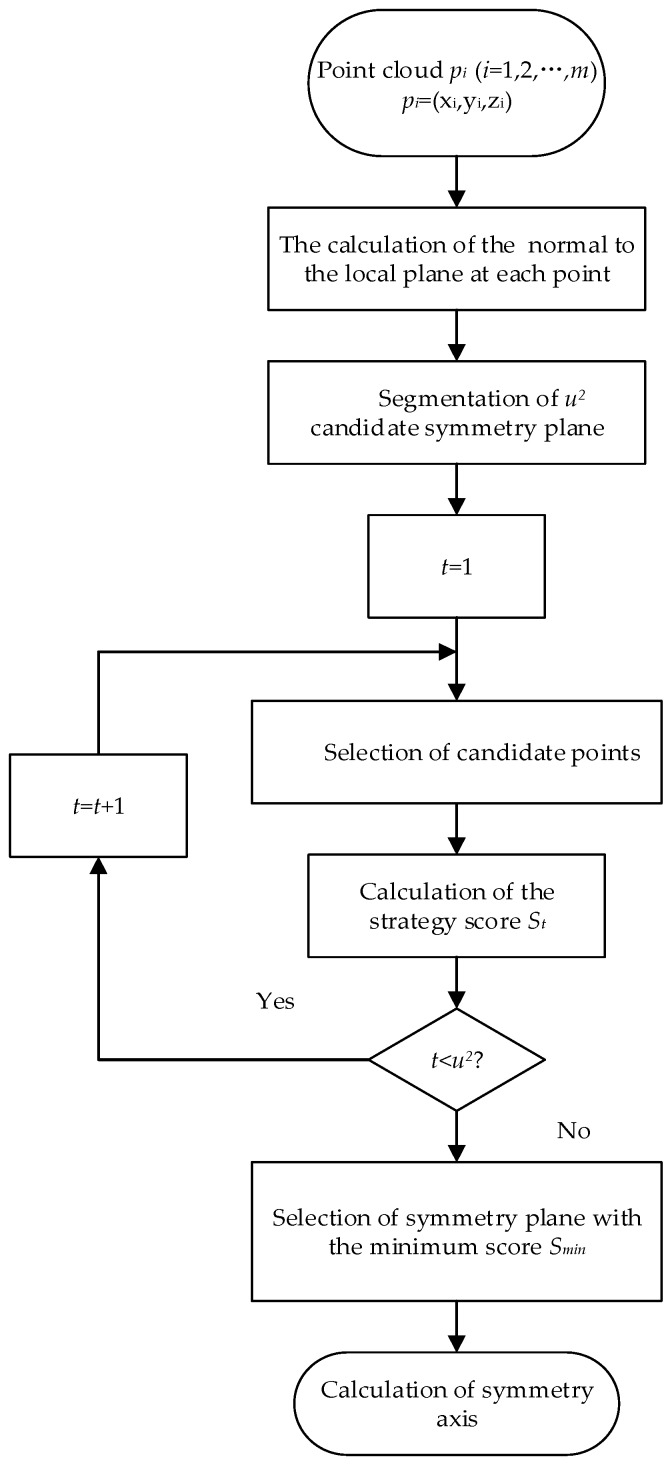
Overview of sweet-pepper pose estimation method.

The symmetry axis, rather than peduncle, should be the focus in developing a sweet-pepper pose estimation scheme. The geometric information in the 3D fruit point cloud was used. In the main part of the algorithm, the normal to the local plane at each point in the fruit point cloud was calculated first. The point cloud was then separated by $u^{2}$ (u > 4, where u is an integer) candidate planes in the spherical coordinate system established with the centroid of the crop (not the centroid of the point cloud) as the origin. A scoring strategy was employed to calculate the scores for each plane separately, and the plane with the lowest score was selected as the symmetry plane of the point cloud. The symmetry axis could finally be calculated using the selected symmetry plane. An overview of our method is illustrated in Figure 4. The different parts of the proposed algorithm are described in the sections below.

### 2.3. Calculation of Normal

The normal is an essential property of point clouds. An estimation of the normal plays an important role in point cloud processing. However, point clouds are prone to containing noise, outliers, and holes because of unavoidable noise, physical errors, and occlusions during acquisition. The three main methods of point-cloud normal-vector estimation are partial surface fitting, the Delaunay/Voronoi method, and robust statistical methods [23,24,25]. Assuming that the sampling plane of the point cloud is smooth everywhere, a local neighborhood of any point can be fitted by the plane. Therefore, a method based on partial surface fitting was used to calculate the normal vector for each point *p* in the sweet pepper cloud. The normal n at a point *p* was calculated by fitting a plane to all the points in the neighborhood of that point. The plane fit was found with the eigenvectors of the covariance matrix *M*. The normal n of the plane was the eigenvector that corresponded to the smallest eigenvalue of the covariance matrix, as follows:

(2) $$M=\frac{1}{k}\overset{k}{\underset{i=1}{\sum }}\left(p_{i}-\bar{p}\right){\left(p_{i}-\bar{p}\right)}^{T},$$

where *k* is the number of neighbors of *p* for fitting a plane; *i* = 1, …, *k*; and $\bar{p}$ is the centroid of *k* neighbor points.

### 2.4. Candidate Symmetry Plane

Before the candidate plane is calculated, the centroid of the fruit $p_{c}$ should be obtained. The weighted average of the coordinate value of each point cannot be used as the centroid of the fruit because the part that is far away from the camera cannot be obtained. The sweet-pepper point cloud was similar to a part of a spheroid. Therefore, the centroid of the sweet pepper could be calculated indirectly. A predetermined number of random points generated based on normal distribution within the vicinity of the original point cloud data were randomly generated. For each random point, the root-mean-square error between each point in the random point cloud and the original cloud data was calculated, as follows:

(3) $$d_{r}=\sqrt{\frac{1}{m}\overset{m}{\underset{i=1}{\sum }}{\left(p_{r}-p_{i}\right)}^{2}},$$

where $d_{r}$ is the root-mean-square error of the distances between each point $p_{r}$ in the random point cloud and the original point cloud data, and *m* is the number of points in the point cloud. Finally, the stochastic point with the smallest root-mean-square error was regarded as the centroid $p_{c}$ of the original point cloud data.

In centering the centroid of point cloud $p_{c}$, a spherical coordinate system was established. The $u^{2}$ (*u* > 4, and *u* is an integer) candidate symmetry planes were then selected using the spherical coordinate system division method, which includes the following steps:

Step 1: The ranges of the horizontal and vertical segmentation angles were defined as φ and θ, respectively. φ had the entire range (φϵ(0,π)), and θ was only for half a sphere ($\theta \epsilon \left(-\frac{\pi }{2},\frac{\pi }{2}\right)$).

Step 2: φ and θ were divided as follows:

(4) $$\varphi_{size}=\frac{\left(\varphi (2)-\varphi (1)\right)}{u},$$

(5) $$\theta_{size}=\frac{\left(\theta (2)-\theta (1)\right)}{u},$$

where $\varphi_{size}$ is the φ variation range, and $\theta_{size}$ is the θ variation range. φ(2) and φ(1) are the maximum and minimum values of φ, respectively. Similarly, θ(2) and θ(1) are the maximum and minimum values of θ, respectively.

Step 3: For each φ, θ was changed *u* times to obtain the $u^{2}$ unit-plane normal vector $n_{p}$, which was calculated as follows:

(6) $$\alpha =(g-1)\cdot \varphi_{size}+\frac{\varphi_{size}}{2}+\varphi (1),$$

(7) $$\beta =(h-1)\cdot \theta_{size}+\frac{\theta_{size}}{2}+\theta (1),$$

(8) x=r⋅sin(α)⋅cos(β),

(9) y=r⋅sin(α)⋅sin(β),

(10) z=r⋅cos(α),

where α is the azimuth in the spherical coordinate system, β is the elevation angle in the spherical coordinate system, *g* is the number of changes in the horizontal segmentation angle, and *h* is the number of changes in the vertical segmentation angle; *x*, *y*, and *z* are the coordinate values of the normal vectors calculated, and *r* is the radius of the unit circle. The point cloud could finally be divided by $u^{2}$ candidate planes.

### 2.5. Calculation of Symmetry Axis

The score of each candidate symmetry plane was calculated according to the preset scoring strategy [26]. Each candidate plane was traversed to determine the one with the lowest score and to select it as the symmetry plane of the target object. The calculation of the score is described below.

Assuming that points are generated from a fluoroscopic imaging device from a single viewpoint, the symmetric partners (the symmetry points of the original points with respect to the candidate symmetry plane) of many points are invisible, and the points without visible symmetry partners are useless for score calculation of the candidate symmetry planes. Therefore, before calculating the score of the candidate symmetry plane, the points without the symmetric partner should be processed.

**Figure 5:**
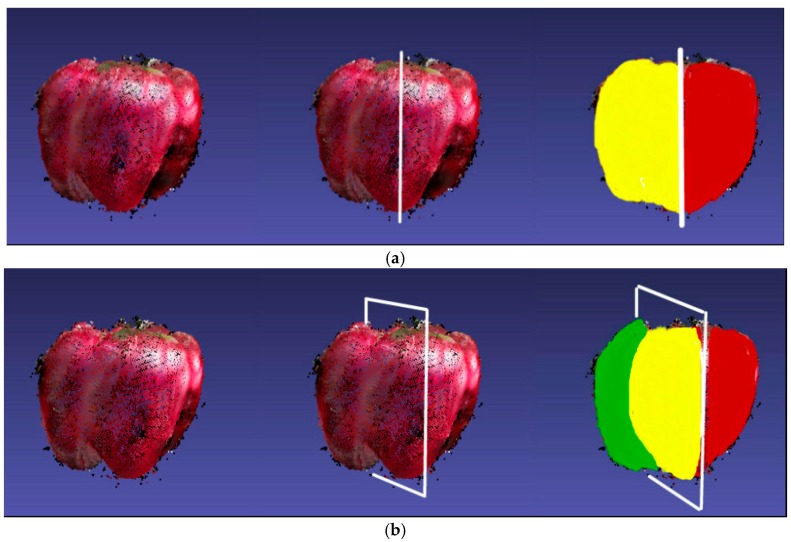
Symmetry planes and visible symmetric partners. For each of the two cases, we show a sweet pepper imaged from a particular point of view (left), estimated symmetry plane (middle, depicted by the intersection of the plane with the sweet pepper), and a color map of points with and without symmetric partners. (**a**) A sweet pepper imaged frontally has virtually all of its points (yellow) in the range data possessing symmetric partners (red). Hence, all points on the left of the symmetry plane are marked yellow; (**b**) a sweet pepper imaged obliquely has the same symmetry plane (now shown rotated). Only the points marked yellow have visible symmetric partners (red), and the green part has invisible symmetry partners.

When viewing an object from one side of a symmetry plane, most of the visible points observed will be those that share the same side with the camera. For the same reason, the points observed from the other side of the camera should have visible symmetry points (Figure 5).

These points were found on the closer side of the candidate plane and the points without partners on the farther side were excluded from the score calculation using surface normals. Point $\dot{p}$ on the surface with an estimated surface normal $n_{x}$ was visible from viewpoint $p_{v}$ if

(11) $$\left(n_{x},p_{v}-\dot{p}\right)>0,$$

where $p_{v}$ is the coordinate of the viewpoint, and the default is (0,0,0). Therefore, points with a symmetric partner and a surface normal that points away from the camera were determined. Point $\dot{p}$ with estimated normal $n_{x}$ was reflected over the candidate symmetry plane with center point $p_{c}$ and normal $n_{p}$ by

(12) $$\ddot{p}=\dot{p}-2\cdot n_{p}\cdot d_{x},$$

where $d_{x}$ is the signed distance between the point $\dot{p}$ and the plane. Correspondingly, $\dot{p}$’s normal was reflected by

(13) $${\tilde{n}}_{x}=n_{x}-2\cdot n_{p}\cdot d_{n},$$

where ${\tilde{n}}_{x}$ is the normal of $\ddot{p},$ and $d_{n}$ is the signed distance between the normal’s head and the candidate plane centered at the camera’s axis origin with normal $n_{p}$. Thus, $\dot{p}$ had no symmetric partner if:

(14) $$\left({\tilde{n}}_{x},p_{v}-\ddot{p}\right)\leq 0.$$

**Figure 6:**
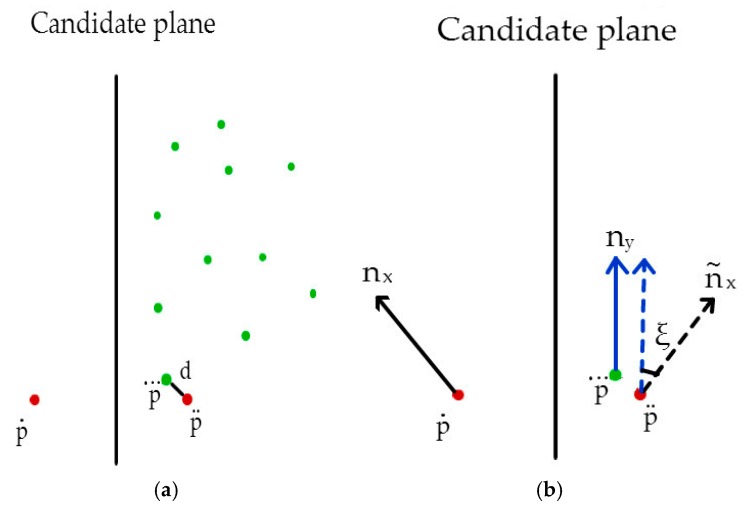
Schematic of reflection score calculation for point $\dot{p}$. (**a**) Determining the closest point $\dddot{p}$ using the reflection point $\ddot{p}$ of $\dot{p}$ and calculating the distance between $\dddot{p}$ and $\ddot{p}$; (**b**) calculating the normal difference ξ between $\dddot{p}$ and $\ddot{p}$.

The score of each candidate point was calculated according to a preset scoring strategy. Specifically, the candidate point $\dot{p}$ was symmetrically transformed with respect to the candidate symmetry plane to obtain symmetrical point $\ddot{p}$. Searching the point with the smallest Euclidean distance from point $\ddot{p}$, point $\dddot{p}$, which is the nearest neighbor to $\ddot{p}$, could be obtained, (Figure 6a). The distance d between $\ddot{p}$ and $\dddot{p}$ and the angle ξ between the normal vector of points $\ddot{p}$ and $\dddot{p}$ were also calculated (Figure 6b).

The score of each candidate point was calculated using Equation (14), and the scores were added as the score of candidate planes; the plane with the lowest score was the symmetry plane.

(15) S=d+τ·ξ,

where S is the score of the candidate point, and τ is the weight of the normal differences relative to the point distances. Parameter τ was determined simply by testing the different values and choosing the best one, which eventually was chosen to be $\frac{3}{\pi }$. The symmetry axis of pepper could be calculated based on the symmetry plane of the point cloud by

(16) $$\gamma =n_{p}\times \frac{p_{v}-p_{c}}{\left|\left|p_{v}-p_{c}\right|\right|},$$

where γ is the symmetric axis vector, $n_{p}$ is the normal vector of the symmetry plane, $p_{v}$ is the coordinate of the viewpoint with a default value of (0,0,0), and $p_{c}$ is the centroid of the fruit. The vector γ starting from $p_{c}$ was considered the symmetry axis of sweet pepper.

## 3. Results and Discussion

### 3.1. Results for Simulation Dataset

**Figure 7:**
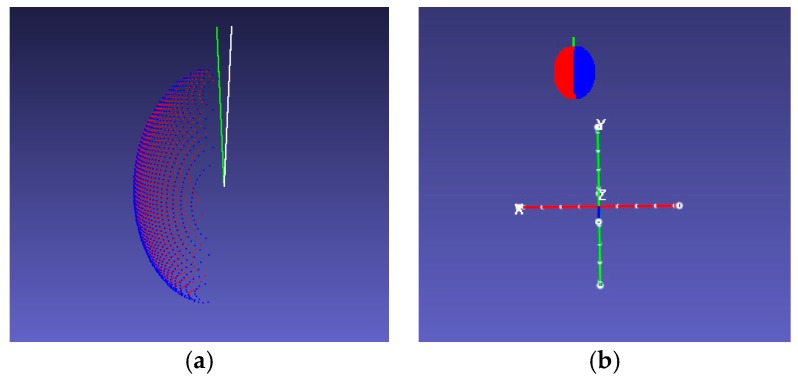
Errors between true and calculated symmetry axes, where the point cloud is rendered in two colors to show the symmetry plane. The green line indicates the calculated symmetry axis, and the white line indicates the real symmetry axis. (**a**) Close-range view; (**b**) camera view.

The symmetry axes of the fitting point cloud were calculated using the proposed algorithm. The results of the point cloud that was 20 cm away from the camera and its true symmetry axes are shown in Figure 7.

**Figure 8:**
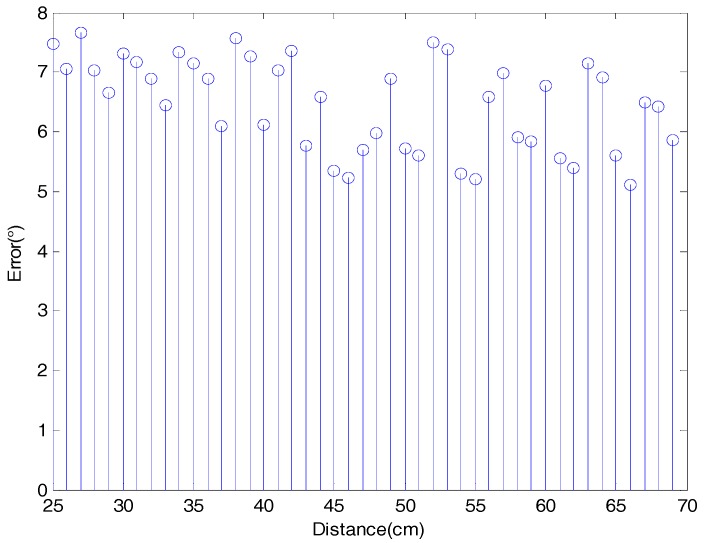
Mean error between real and calculated axes for each distance of the simulated point cloud from the camera.

The presented method was tested based on 45 point clouds 50 times. The mean errors between the two axes in the simulated experiments are shown in Figure 8, where the average error was 6.4710°.

Figure 8 shows that the error for each distance between the real and the calculated symmetry axis varied between 5° and 8° as the distance between the point cloud and the viewpoint increased. Given that the distance of the fruit point cloud from the camera was not more than 70 cm when using the Intel RealSense SR300, the upper limit distance of the simulation experiment was set to 70 cm. From a distance change of 25 cm to 70 cm, the average deviation of each distance does not vary by more than 3° because the angle is invariant for the perspective projection and should not change with increasing distance from the camera. Therefore, although the change in distance causes reduced accuracy of the point cloud obtained from the camera, it has no effect on the proposed algorithm for pose estimation.

### 3.2. Results for Sweet-Pepper Dataset Analysis

**Figure 9:**
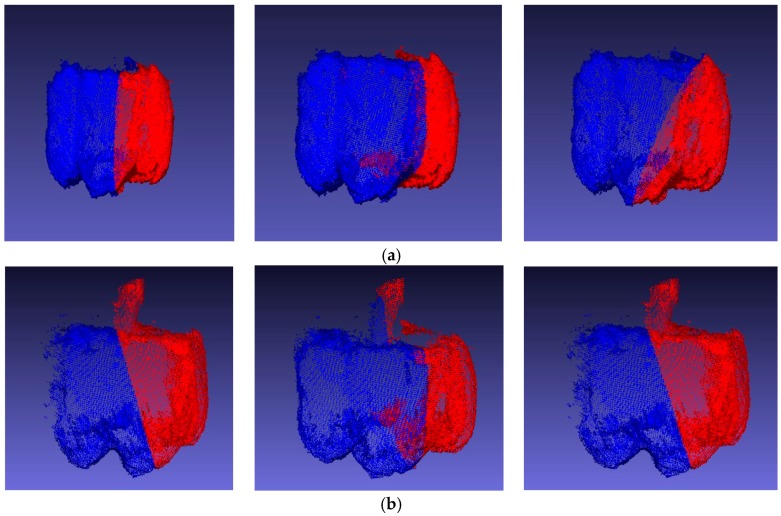
Six effects of $u^{2}\;$ segmentation. The sides of the candidate plane are shown in red and blue. (**a**) The left picture shows the symmetry plane of the point cloud without peduncle; (**b**) the left picture shows the symmetry plane of the point cloud with peduncle.

The normal vector of each point in the point cloud could be directly calculated because the data were prefiltered. The sweet pepper cloud was separated from (u = 6) planes using the methods mentioned in the previous section (Figure 9). The score of each plane was calculated.

**Figure 10:**
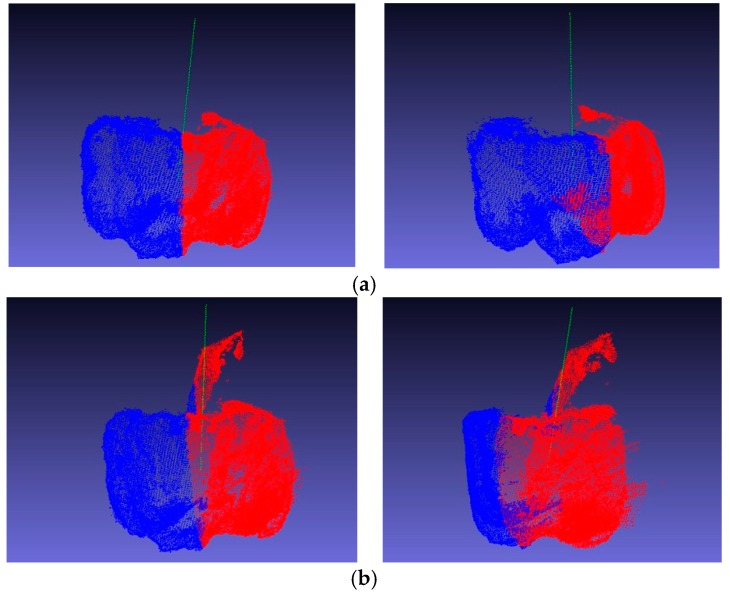
Sweet-pepper cloud with calculated symmetry axes in different perspectives, where the green dotted lines indicate symmetry axes. (**a**) Sweet pepper without peduncle; (**b**) sweet pepper with peduncle.

The best plane was selected as the symmetry plane of the point cloud. The axis was calculated based on the obtained symmetry plane (Figure 10).

**Figure 11:**
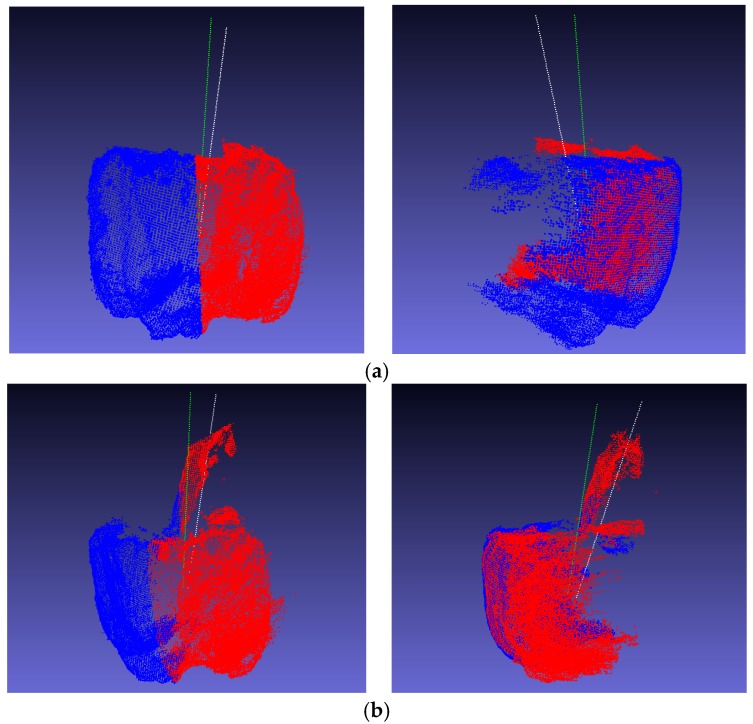
Calculated symmetry axes and the peduncle with standard symmetry axes from different views, where the green lines indicate the calculated symmetry axes, and the white lines indicate the real symmetry axes. (**a**) Sweet pepper without peduncle; (**b**) sweet pepper with peduncle.

The real symmetry axis could not be obtained because the sweet-pepper point cloud provided by Sa et al. was not manually calibrated. The fruit peduncle was used to calculate the symmetry axis of the sweet pepper in our experiments. The weighted average of the point cloud coordinates of the peduncle cloud was first considered as the center of the peduncle. The extension line of the center of the peduncle and the center of the fruit in the sweet-pepper point cloud experiment was considered to be the true axis of symmetry. The qualitative results are shown in Figure 11.

**Figure 12:**
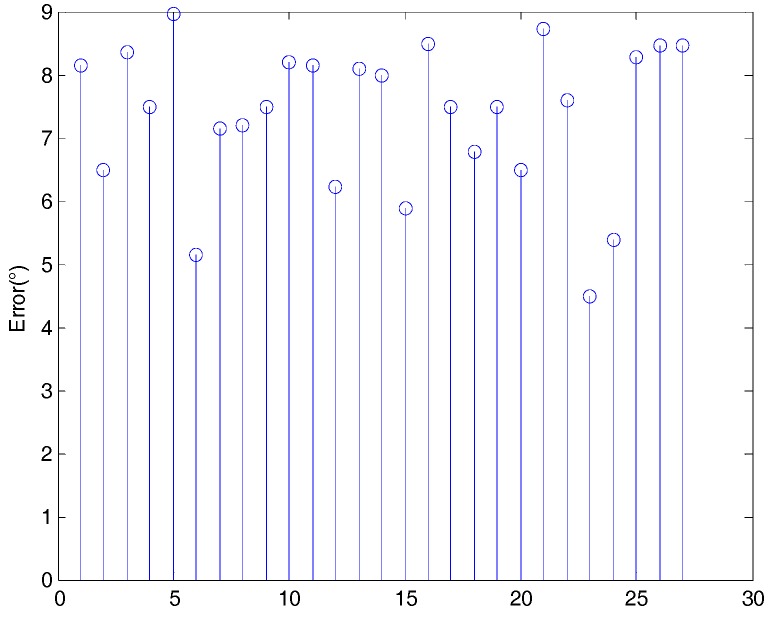
Errors between calculated and real symmetry axes when the peduncle was removed.

The error between the two axes of experiments on sweet pepper without peduncle are shown in Figure 12. The error ranged from 3.6957° to 9.3433°, and the average error was 7.3729°.

**Figure 13:**
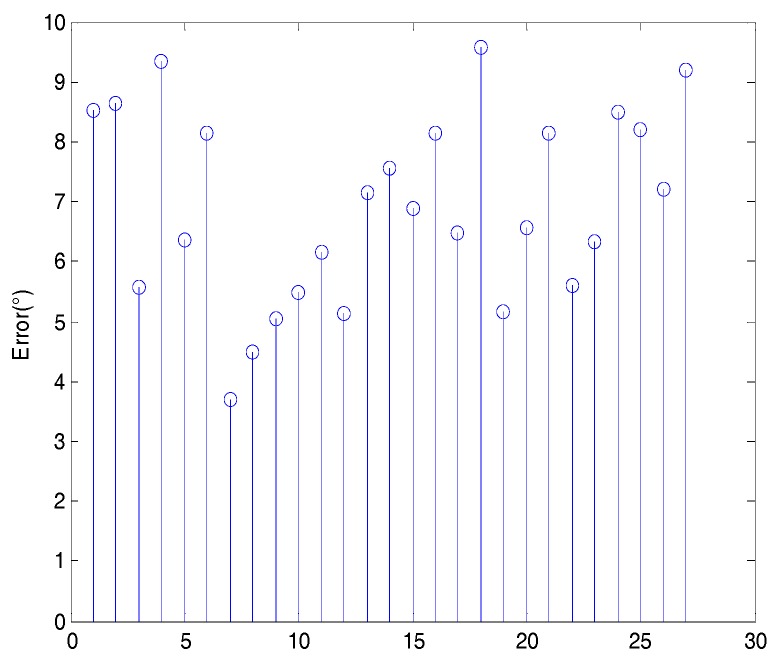
Errors between calculated and real symmetry axes when the peduncle is complete.

When the peduncle was complete, the quantitative results shown in Figure 13 were obtained. The error ranged from 4.4879° to 8.9578°, and the average error was 6.9343°.

Figure 12 and Figure 13 show the slightly different results of the experiments using the sweet-pepper point cloud with peduncle and without peduncle. When the peduncle was complete, the average error of the experiment was 6.9343°, which was lower than the average error (7.3729°) of the experiment on sweet pepper without peduncle. The sweet pepper with peduncle allowed obtaining more geometric information than the sweet pepper without the peduncle.

### 3.3. Discussion

The results of sweet-pepper point cloud analysis and experiments of the simulated point cloud were compared. The error based on the sweet-pepper point cloud was larger than the average based on the simulated data. On one hand, the standard axis of symmetry in the experiment of simulating a point cloud was determined by the ellipsoid equation, and the extension line of the center of the peduncle and the center of the fruit in the sweet-pepper point cloud experiment was artificially regarded as the standard symmetry axis. This finding may affect the experiment error calculation on the sweet-pepper point cloud. On the other hand, the simulated point cloud was more regular than the shape of the sweet-pepper point cloud. Despite the error, the proposed method for detection of the symmetry axis could still estimate the pose of sweet pepper when the peduncle was invisible and visible.

Fruit pose estimation through the direct detection of peduncles using machines remains challenging because peduncles are small and often occluded by the fruit. In contrast to other methods, the proposed pose estimation method does not depend on the peduncle of the sweet pepper. Given that the shape of the sweet-pepper point cloud is more complex than those of other fruits, such as apples, pose estimation, applied here to sweet pepper, will be easier when the algorithm is applied to fruits with regular shapes.

In this study, the pose of non-occluded sweet pepper was estimated. To improve the accuracy when the fruit is occluded, future research should focus on two aspects: firstly, the point cloud without serious occlusion (the occlusion area accounts for less than one-half of the cross-sectional area of the fruit) can be fitted by least squares [27], and the approach can be improved based on the fitted point cloud; secondly, seriously occluded sweet pepper (the occlusion area accounts for more than one-half of the cross-sectional area of the fruit) can be detected and labeled, such that the robot would perform localization and movement planning, and thus, prioritize normal sweet peppers for harvesting.

## 4. Conclusions

This study proposed a machine vision approach to estimate the pose of sweet pepper based on its symmetry axis. The method can also overcome the case where the peduncle is too thin to detect and when self-occlusion of the peduncle occurs. In the simulated test, the mean angle error between the calculated symmetry axis and the real axis was approximately 6.5°. In a particularly challenging sweet-pepper cloud dataset test, the average error was approximately 7.4° when the peduncle was removed. When the peduncle of sweet pepper was complete, the average error was approximately 6.9°. The proposed method can not only estimate the pose of fruit to provide effective guidance for the end-effector in the detachment process, but can also be used to detect the symmetry axis of regular objects. However, the improvement required for the presented approach is remarkable, especially for the occluded fruit. For point clouds without serious occlusion, the improvements can be made based on the fitted point cloud by least squares. For seriously occluded sweet pepper, the robot would perform localization and movement planning to prioritize normal sweet peppers for harvesting.
